# Rowing Simulator Modulates Water Density to Foster Motor Learning

**DOI:** 10.3389/frobt.2019.00074

**Published:** 2019-08-21

**Authors:** Ekin Basalp, Laura Marchal-Crespo, Georg Rauter, Robert Riener, Peter Wolf

**Affiliations:** ^1^Sensory-Motor Systems Lab, Department of Health Sciences and Technology, Institute of Robotics and Intelligent Systems, ETH Zurich, Zurich, Switzerland; ^2^Motor Learning and Neurorehabilitation Laboratory, ARTORG Center for Biomedical Engineering Research, University of Bern, Bern, Switzerland; ^3^BIROMED-Lab, Department of Biomedical Engineering, University of Basel, Basel, Switzerland; ^4^Reharobotics Group, Spinal Cord Injury Center, Balgrist University Hospital, Medical Faculty, University of Zurich, Zurich, Switzerland

**Keywords:** robot-assisted training, motor learning, practice variability, functional task difficulty, contextual interference, augmented feedback, sports engineering

## Abstract

Although robot-assisted training is present in various fields such as sports engineering and rehabilitation, provision of training strategies that optimally support individual motor learning remains as a challenge. Literature has shown that guidance strategies are useful for beginners, while skilled trainees should benefit from challenging conditions. The Challenge Point Theory also supports this in a way that learning is dependent on the available information, which serves as a challenge to the learner. So, learning can be fostered when the optimal amount of information is given according to the trainee's skill. Even though the framework explains the importance of difficulty modulation, there are no practical guidelines for complex dynamic tasks on how to match the difficulty to the trainee's skill progress. Therefore, the goal of this study was to determine the impact on learning of a complex motor task by a modulated task difficulty scheme during the training sessions, without distorting the nature of task. In this 3-day protocol study, we compared two groups of naïve participants for learning a sweep rowing task in a highly sophisticated rowing simulator. During trainings, groups received concurrent visual feedback displaying the requested oar movement. Control group performed the task under constant difficulty in the training sessions. Experimental group's task difficulty was modulated by changing the virtual water density that generated different heaviness of the simulated water-oar interaction, which yielded practice variability. Learning was assessed in terms of spatial and velocity magnitude errors and the variability for these metrics. Results of final day tests revealed that both groups reduced their error and variability for the chosen metrics. Notably, in addition to the provision of a very well established visual feedback and knowledge of results, experimental group's variable training protocol with modulated difficulty showed a potential to be advantageous for the spatial consistency and velocity accuracy. The outcomes of training and test runs indicate that we could successfully alter the performance of the trainees by changing the density value of the virtual water. Therefore, a follow-up study is necessary to investigate how to match different density values to the skill and performance improvement of the participants.

## Introduction

In recent years, developments on computer processing capabilities, and robotic systems have given rise to robot-assisted training in many fields, e.g., in rehabilitation (Marchal-Crespo and Reinkensmeyer, 2009), in sports simulation (Rauter et al., 2019) and in surgical training (Enayati et al., 2018). Such robotic systems used in various domains share the common purpose of supporting humans improving/acquiring new skills. Thus, established principles and theorems from the field of motor learning become invaluable tools to be employed by such robotic systems.

Motor learning is perceived to be a problem-solving process (Guadagnoli and Lee, 2004). When humans attempt to learn a new skill, receiving information regarding their performance during training becomes crucial, since it helps choosing the correct action plan for solving the problem (Miller et al., 1960). In general, availability of information promotes the rate of motor learning and the quality of the performance. According to the challenge point framework, learning is dependent on the available and individually interpretable information during training, which is related to the functional task difficulty. The framework describes the functional task difficulty as the difficulty of the task relative to the skill level of the learner and the conditions under which the task is practiced. When the functional task difficulty is matched to the individual skill level, i.e., the entire information can be interpreted, the challenge point is achieved and therewith, motor learning is optimally promoted (Guadagnoli and Lee, 2004). The functional task difficulty can be adjusted in terms of feedback information and contextual interference to match the individual skill level.

Certain studies in the domain of robot-assisted sports training investigated augmented feedback designs that could target different skill levels. Novice participants were observed to benefit from haptic error reduction in golf (Duarte and Reinkensmeyer, 2015), haptic guidance in tennis (Marchal-Crespo et al., 2013), and various unimodal as well as multimodal feedback designs in a complex rowing task (Rauter et al., 2015; Sigrist et al., 2015). Naïve participants' motor learning was also investigated by adapting the provided feedback to the participants' performance in the rowing task (Rauter et al., 2019). The study successfully showed the benefits of automated and individualized feedback selection for beginners; however, further investigation is needed to extend the findings to more advanced participants. Although challenging feedback has been shown to have a positive effect on upper limbs (Patton et al., 2006; Milot et al., 2018) and lower limbs (Reisman et al., 2013; Marchal-Crespo et al., 2017) with robotic rehabilitation systems, only few studies investigated the application of challenging feedback designs on advanced participants, all of which showed ineffective results to date. Haptic error augmentation in golf (Duarte and Reinkensmeyer, 2015), resistive forces to correct the performance in rowing (Rauter et al., 2015) did not benefit participants, which might be attributed to inadequate skill level of the learners and demotivational effects of the challenging feedback designs. Our team also designed a visual error amplification feedback (Basalp et al., 2016), which later targeted non-naïve participants for the rowing task (Gerig et al., 2019). However, we could not find any effectiveness of visually augmented error for the complex sports task. Thus, it remains an open task in robot-assisted sport training to design challenging methods that support learning from early to late motor skill stages.

Robot-assisted training offers many possibilities to modulate the information available for the learner. Robot-assisted training can feature a haptic interface physically interacting with the user. The haptic interface can render task-specific forces, e.g., water resistance in a rowing simulator (Rauter et al., 2013). Thus, instead of providing the information as an augmented feedback design, robots may also allow modulation of task kinematics and dynamics to assist learning.

Modulation of task characteristics can yield different levels of functional task difficulty on a given nominal task. More precisely, the modulation of task characteristics can yield different conditions under which the task is performed, which alters the difficulty perceived by the learner. In a recent study, this effect was explained by the term “c*onditional task difficulty*” as the difficulty of the task relative to the task conditions (Baur et al., 2018). Although the scope of conditional task difficulty is encompassed by the functional task difficulty, the former term extends the definition the latter such that the challenge resulting from the task conditions (motor task aspect) and skill level of learner (human aspect) are distinguished. This clarification is important because the robotic systems can directly modulate the conditional task difficulty while skill level is more dependent on the capacity and ability of the learner.

As explained in the challenge point framework, the difficulty should be optimally adapted to assist the learners benefit from the resulting potential information to learn the task. Nevertheless, in many cases with complex tasks, there is no a priori information on how to optimally adapt the difficulty to the participant's skill level. Thus, methods that induce contextual interference effects can also be employed to modulate the amount of challenge (Guadagnoli and Lee, 2004).

In motor learning, the contextual interference effect is defined as the interference resulting from the fact that many tasks are practiced all together within the same training session (Magill and Hall, 1990; Lee et al., 1992). The effect of contextual interference on learning has been shown in studies that investigated practice schedule, i.e., random vs. blocked training (Sherwood, 1996; Wright and Shea, 2001; Guadagnoli and Lee, 2004). In this type of studies, the variations of a task are practiced in either blocks of one task type (blocked practice; e.g., AAA-BBB-CCC) or in blocks of varying types of task (random practice; e.g., ACB-BAC-BCA) (Akizuki and Ohashi, 2013). However, if the chosen conditions (i.e., A, B, and C) are not carefully designed within the blocked practice, progression from one block to the next one may not match the skill development of the learner.

The contextual interference can also be considered from the perspective of “practice variability,” i.e., variable vs. constant training (Schmidt, 1975). Variable training refers to the situation that a learner practices multiple variants of a task in a training session. Various theorems (elaboration hypothesis: Shea and Morgan, 1979; reconstruction hypothesis: Lee and Magill, 1983; schema theory: Schmidt, 1975) in motor learning attribute the effectiveness of both the randomization of schedule and practice to the increased cognitive activity while attempting to learn the tasks. Thus, contextual variability can foster the learning and transfer of practiced skills (Shea and Morgan, 1979; Wymbs et al., 2016). In literature, positive effects of the increased contextual variability was seen in ball throwing (Elfaqir, 1982), soccer (Williams, 1998), tennis (Hernández-Davo et al., 2014), baseball (Hall et al., 1994), and basketball (Memmert, 2006).

Therefore, in this study, we investigated the effect of modulation of inherent task characteristics on a real-life complex rowing task. The practice variability was imposed by modulating the density of the simulated water in our rowing simulator. We assumed that the change of water resistance forces would increase the conditional task difficulty; hence, the functional task difficulty, when presented in a randomized order within the training blocks. Thus, we hypothesized that the group that trained with variable density conditions would show superior learning and transfer compared to the group that trained with fixed density condition.

## Methods

### Participants

Sixteen healthy naïve (non-rower) participants (8 females, 8 males; age range = 19–38 years; mean age 24.9 years) were recruited. Inclusion criteria were normal hearing and normal or corrected-to-normal vision, no experience with the task, and at least half an hour of exercise per week. All participants signed an informed consent following the guidelines of the ETH Zurich Ethics Commission, which had approved the study (EK 2017-N-27). Participants were verbally instructed about the experimental procedure along with the risks, and the possibility of withdrawal from the study at any time without providing further reasons or dealing with consequences. Participants were randomly assigned to either control or experimental groups in a single blinded fashion.

### Setup

**Figure 1:**
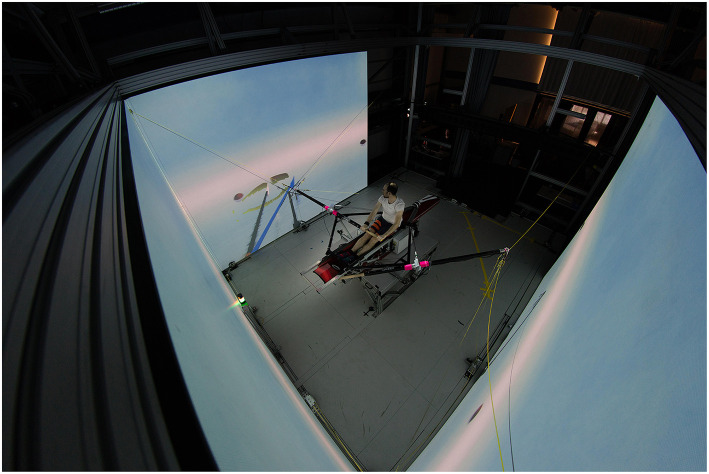
Custom built rowing simulator with a trimmed single scull boat in the middle and screens surrounding it. The person sitting in the boat is the first author of the paper. He demonstrates the visual scenario and the augmented visual feedback that is provided during the training sessions.

For the study, our custom-built rowing simulator was used (Rauter et al., 2010; see Figure 1). The rowing simulator is composed of a trimmed single scull boat (Stämpfli Racing Boats AG, Zürich, Switzerland) that is set up in the middle of three 4.4 m × 3.3 m screens placed in front of the stern and on each side of the boat (von Zitzewitz et al., 2008).

Three projectors (Projection Design F3+, Norway) displayed the visual scenario on the screens including an ocean scenario, concurrent augmented visual feedback during training, and a performance chart after non-feedback trials. Visuals were developed in Unity (Unity Technologies ApS, CA, USA). The minimum update rate was set to 30 fps.

Auditory rendering of the virtual water-oar interaction was developed in C++. Sounds were delivered by speakers (DELL A525 Zylux Multimedia Computer Speaker System) with an update rate of 30 Hz.

Haptics of the task were displayed by a tendon-based parallel robot (Rauter et al., 2010) which actuated a shortened oar. The control of the robot was done by a Matlab/Simulink® model (r2013b, The MathWorks, Inc., MA, USA) running on an xPC-target at a fixed update rate of 1,000 Hz. In the instruction session, the displayed haptics were the output forces of a PD based position control (Rauter et al., 2015) that fully guided the participant through the oar movement (i.e., haptic guidance). In the remaining part of the study protocol, water resistance forces calculated from the virtual water-oar interaction were rendered.

### Task

In this study, a trajectory-reproducing rowing task was chosen. The task was the same as in our previous studies (Rauter et al., 2015, 2019; Basalp et al., 2016; Gerig et al., 2019). Participants performed a trunk-arm sweep rowing at the port side (right) of the boat. In sweep rowing, rowers manipulate a single oar with both hands. Trunk-arm rowing is usually executed in rowing trainings as a way to improve technique and team coordination, as a warm-up exercise, and as the technique used in para-rowing. Trunk-arm rowing can be categorized as a continuous rhythmic motor task (Marchal-Crespo et al., 2015).

Participants were asked to keep the oar blade in squared (vertical) orientation, i.e., the blade rotation in the longitudinal axis was omitted in the task. Trunk-arm rowing was executed mainly with coordinated arm and torso flexion/extension movements. The feet were placed in the shoes, legs were kept stretched. To account for different leg lengths, the position of the foot-stretcher was adjusted. The boat in the simulator was fixed onto a platform and roll angle was kept constant due to safety requirements. Thus, the participants did not need to correct for the roll angle, i.e., balance of the boat.

Although the leg drive and blade rotation are excluded, trunk-arm rowing is a real-world complex task since it requires several degrees of freedom and cannot be mastered in one session due to the fast changing oar interaction dynamics between the air and water (Wulf and Shea, 2002). One rowing stroke is composed of four phases that incorporate distinct kinematic and dynamic characteristics. These phases are called catch, drive, release and recovery. The drive phase is where the oar blade is fully in the water; and the recovery, where the blade is moving in the air. The catch and release phases are the transition from air to water and vice versa, respectively.

The trunk-arm rowing task was shown to the participants by means of a reference oar blade trajectory. The reference trajectory was recorded from an expert rower and further processed to result in a smooth and cyclic C^2^ continuous trajectory. Exact duration of one stroke was calculated to be 2.5 s, i.e., 24 strokes per minute (spm). The resultant trajectory was resized to a suitable movement range at the oar handle: A horizontal span of 0.67 m (44° horizontal oar angle, i.e., θ, at the oarlock) and a vertical span of 0.19 m (12.5° vertical oar angle, i.e., δ), which was fixed for all participants. The reference trajectory was presented by means of position control in the instruction session. During position control mode, participants held the oar handle while the rowing simulator haptically controlled the oar position (Gerig et al., 2019). Thus, the participants passively followed the controlled oar to observe the desired spatial and velocity profile of the reference trajectory. In the rest of the protocol, the reference was visually shown to the participants on the right hand side screen. The main task of the participants consisted of reproducing the reference trajectory as accurately as possible by paying attention to its spatial and velocity profiles. The task and instructions to the participants were the same as in prior studies on our rowing simulator (Rauter et al., 2015; Sigrist et al., 2015; Gerig et al., 2019).

### Re-modeling of Rowing Task Haptic Rendering

The effect of the modulated conditional task difficulty was explored on the haptics of the rowing simulator. To realize this, a rowing model, whose variables can be directly controlled with our control graphical user interface, was required to render and modify the oar blade—water interaction characteristics. We changed the force model of rowing simulator that was previously described in (von Zitzewitz et al., 2008), Rauter et al. (2010). In the previous model, drag (*C*_*D*_), and lift (*C*_*L*_) coefficients that yield the drag (*F*_*D*_) and lift (*F*_*L*_) forces on the oar blade, were approximated by a function of angle of attack and a constant term called as maximal oar lift coefficient ($C_{O_{L}}^{\max}$). To parametrize the calculation of *F*_*D*_ and *F*_*L*_, instead of a predefined constant of $C_{O_{L}}^{\max}$, the formula suggested by Caplan and Gardner (2007) was used for the updated rowing model as follows:

(1) $$\begin{array}{r} F_{D}=\frac{1}{2}C_{D}\rho A_{D_{p}}V_{O/w}^{2} \end{array}$$

(2) $$\begin{array}{r} F_{L}=\frac{1}{2}C_{L}\rho A_{L_{p}}V_{O/w}^{2} \end{array}$$

where ρ is the density of the water; *V*_*O*/*w*_ is the relative velocity between oar blade and water; and *A*_*L*_*p*__ and *A*_*D*_*p*__ are the projected areas of the oar blade for lift and drag forces, respectively.

In this mathematical model, the drag force acts in the opposite direction of the *V*_*O*/*w*_ and the lift force acts perpendicular to it. Calculation of both *C*_*D*_ and lift *C*_*L*_ were performed with a look-up table whose input was the angle of attack (α) as proposed in Caplan and Gardner (2007).

Calculation of projected areas were based on a function that was dependent on the oar angles and properties of the oar:

(3) $$A=f(\delta ,\;\theta ,\;\varphi ,\;l_{ob},\;h_{ob},\;l_{oar})$$

where φ is the longitudinal oar angle; *l*_*oar*_ is the length of the oar from oarlock to the end of blade and *l*_*ob*_ and *h*_*ob*_ are the length and height of the oar blade. *V*_*O*/*w*_ was calculated from the oar angles and the boat velocity *V*_*b*_:

(4) $$V_{O/w}=f(\delta ,\;\theta ,\;\varphi ,\;l_{oar},\;V_{b})\;$$

**Figure 2:**
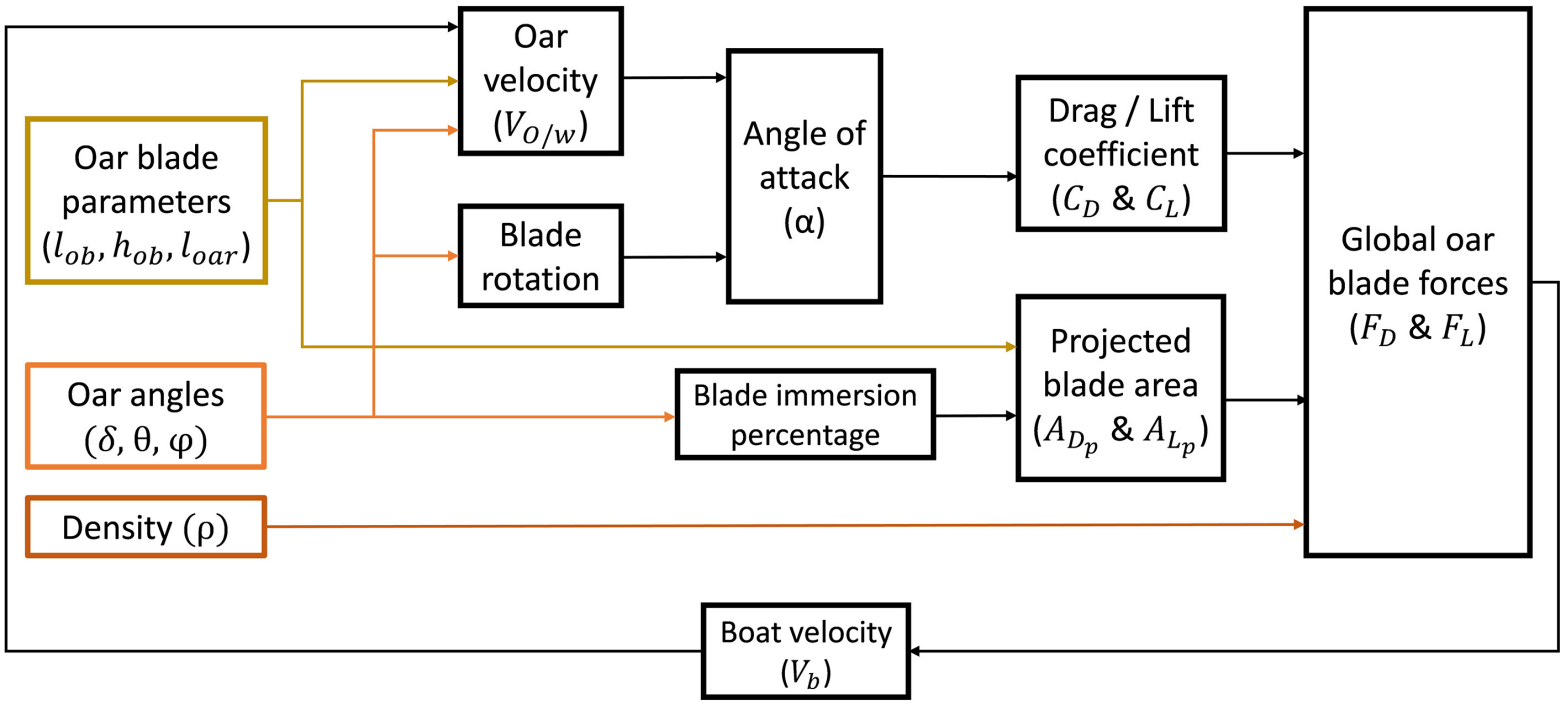
Flow chart of the angle-dependent water interaction model. Oar angles (δ, θ, φ) were received from sensors. Oar blade parameters were adapted from von Zitzewitz et al. (2008) and the value of density (range 50–4000 $\frac{kg}{m^{3}}$) was set from the control GUI.

The modulation of the water density had a direct effect on the perceived resistive task forces, i.e., *F*_*D*_ & *F*_*L*_ (see Figure 2). Since the participants were asked to keep the oar blade in squared orientation, change of drag forces in the horizontal plane had an impact on the temporal aspect (i.e., magnitude of velocity) of the task, mainly in the late catch, all drive phase and the early release. Additionally, the change in the lift forces in the vertical plane affected the spatial aspect of the movement, especially in the catch and release phases where the air-water transition takes place. In the study, six different density values were chosen (see Table 1).

**Table 1:** Selected density values for haptic rendering of different virtual water conditions: Water density characteristics column lists the magnitude of perceived water resistance forces.

**Condition and density ($\frac{kg}{m^{3}}$)**	**Water density characteristics**	**Water color in visual scenario**	**Provided session**
A: ρ = 200	Very low	Purple	Baseline and transfer
B: ρ = 400	Low	Orange	Training for VD
C: ρ = 1100	Normal	Blue	Baseline, training and retention
D: ρ = 1800	Above-average	Red	Baseline and transfer
E: ρ = 2500	High	Green	Training for VD
F: ρ = 3200	Very high	Yellow	Baseline and transfer

*Water color in visual scenario column lists the visual rendering of the water scenario on the screens*.

The nominal water density (i.e., nominal task condition) was selected as ρ = 1100 (Condition C in Table 1). The short- and long-term motor learning was assessed with this nominal condition in the retention tests. For the transfer tests, A, D, and F were chosen to be able to generalize the outcome to the lowest, above-average and highest density conditions. During training sessions with visual feedback, the conditions B, C, and E were provided in a prefixed random order.

### Experimental Protocol

Each participant was asked to come to the laboratory on three consecutive days. The study was designed in a between-participants fashion with two groups: Variable (VD) and Fixed Task Difficulty (FD), which served as the experimental and control group, respectively. A total of 16 participants were equally assigned to each group in a random but gender-balanced manner, i.e., there were 8 participants for VD (4 females, 4 males, 19–33 years, mean age 25.3 years) and 8 participants for VD (4 females, 4 males, 19–38 years, mean age 24.6 years).

**Figure 3:**
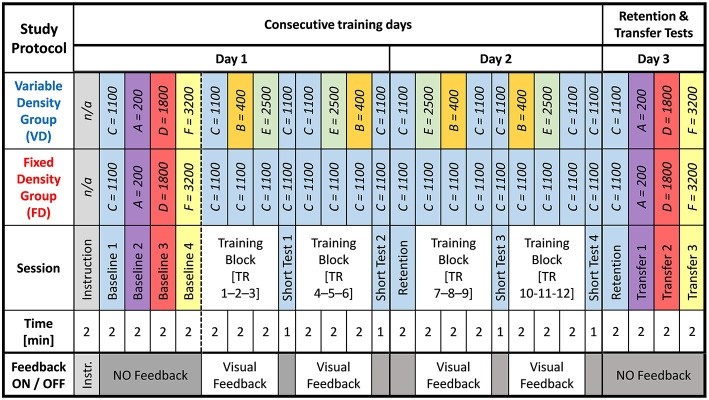
Study protocol showing the groups (VD and FD) and received training methods: The density values that were presented in each training or test session were shown with the corresponding letters, e.g., *C* = 1100 is *ρ* = 1100 $\frac{kg}{m^{3}}$.

The control group (FD), and experimental group (VD) differed in the density conditions employed during training. While FD was subject to a series of training sets with fixed nominal density (Condition C, Table 1), the VD group received a previously adjusted, equally balanced random order of density conditions (B, C, and E, Table 1), as shown in Figure 3. The modulation of the density conditions were independent of the VD group participants' performance. All the VD group participants received the same random order of density conditions (Figure 3).

In the beginning of Day 1, participants were informed about the protocol and the goal of the study, risks and safety measures of the rowing simulator and the participation rights. After verbal briefing, the study investigator demonstrated the reference task in the boat under the guidance of position control while the participant observed him. During this initial basic instruction, participants were informed about the interaction with the oar and the relevant task kinematics.

Following the basic instruction, participants were asked to sit in the boat and perform the same reference trajectory under the full robotic guidance with the position control for 120 seconds (s). This served as a hands-on instruction that showed the participants the handling of the oar and the spatial and velocity profile of the reference stroke. In total, 48 reference strokes were performed. The importance of this comprehensive instruction session was to familiarize the participants with the use of our complex rowing simulator, so that they could confidently attempt performing the task by themselves. All the participants were instructed by the same study instructor to particularly pay attention to the spatial and temporal aspects of the reference stroke movement, since they would be asked to reproduce this movement as accurately as possible in the baseline test runs, in which no external information about their performance would be given. Besides the robotic guidance, participants were not given any additional hints on how they should use this information. Although the oar movements took place on the right hand side of the participants, they were free to develop their own strategies such as looking straight ahead to the front screen or watching the guided oar blade movement on the right.

The baseline tests were done after the instruction session. In total, four baseline tests with different water densities, each of 120 s long, were performed. The first baseline test was always performed under the density condition C, i.e., nominal condition. The order of the following three tests (A, D, and F) were randomly selected out of six possible combinations by rolling a dice for each participant. During these four baseline tests with different density conditions, participants tried to reproduce the reference trajectory from what they could recall from the instruction session. Only the visuals of an ocean scenario, oar movement, boat and the buoys showing the rowing lane boundary were rendered on the screens. The displayed water color on the screens was changed according to the chosen density condition (see Table 1). In terms of haptics, only the water-oar interaction forces were rendered. For the auditory rendering, only the water interaction and splash sounds were displayed from the speakers. No additional information or feedback related to the reference trajectory was provided. The same configuration of haptic, visual and auditory rendering was persistent over all baseline, retention and transfer tests.

After the baseline tests, the training session took place. Each training session was designed in blocks of three training sets (duration: 120 s each) followed by a short-term retention test (No-feedback trial; duration: 60 s). In total, there were six training sets and two no-feedback trials (Figure 3). During the training sets, both groups were given the same reference trajectory in terms of a concurrent augmented visual feedback on the right hand side screen (Sigrist et al., 2015; Gerig et al., 2019). The visual feedback was not present in the no-feedback trials, neither in the baseline, long-term retention (RE2 and RE3) and transfer tests.

On Day 2, participants were asked to perform a long-term retention test (RE2) with the nominal density condition C. The rest of the training session was identical to Day 1, except the order of the density conditions that the VD group was trained with.

On Day 3, the final long-term retention test (RE3) and the following three transfer test conditions were performed in the same way as described for Day 1.

Whenever the density condition was modulated for the tests and training, the participants were not explicitly informed if the density was increased or decreased. They were only told that each density condition would correspond to a specific water color that was visually rendered on the screens. Thus, the exact change of the task forces were not readily predictable before the participants started rowing.

Before starting the baseline, retention and transfer tests, all the participants were instructed to reproduce the spatial and velocity profile of the reference movement as accurately as possible. The only additional interference to the participants' performance was a verbal warning if they executed three successive cycles outside the predefined range of rowing stroke rate, i.e., 22–26 spm. This interference aimed to clear all possible effects on performance induced by a speed-accuracy trade-off (Gerig et al., 2019).

After completion of each test and session, i.e., baseline, training, retention, and transfer, participants were asked to complete an Intrinsic Motivation Inventory (IMI) questionnaire to obtain insights about their perceived competence, effort, interest and task usefulness (see Intrinsic Motivation Inventory Questionnaire).

After the completion of each test, i.e., baseline, no-feedback trial, retention and transfer tests, participants from both groups were shown their mean spatial and velocity error values on a 2D line chart on the front screen (see Setup). This performance chart was used to provide knowledge of results (KR) and it showed the progress of each participant's accuracy from the first to the last performed test across days. After each test, participants were able to look at their own performance chart as long as they wanted and compare the current performance with the previous tests. Besides what the participants grasped from their own assessment from the performance chart, they were not given any further strategy from the study instructor.

### Intrinsic Motivation Inventory Questionnaire

The IMI questionnaire is a type of qualitative measurement tool, which was designed to evaluate the participants' subjective perception regarding the activity performed in an experiment (Ryan and Deci, 2000). It is used as a multidimensional assessment (with six subscales) for determining the perceived choice, interest, competence, effort, usefulness and pressure for a given task.

In this study, the IMI was modified to include only the more relevant subscales for this specific experimental task: interest/enjoyment, perceived competence, effort, and the value/usefulness. Each subscale, except the usefulness, was assessed with one normal and one reverse question (negative). The order of the seven questions was randomized and the resulting list of questions was used after each test and training set. Participants were given a pen and asked to fill the IMI by themselves. Before answering the questionnaire, participants could see all of their previous answers but the study instructor was not able to see them until the end of protocol.

### Outcome Metrics

In order to facilitate the understanding of the presented results, we first define the terms *motor performance, motor learning*, and *transfer*.

In this paper, we consider *motor performance* as the movement error and variability during training trials, in which participants performed the task under the guidance of concurrent visual feedback. *Motor learning* is regarded as the change in movement error and variability from the baseline to long-term retention tests. Therefore, motor performance was associated with instant and temporary changes in performance due to the influence of augmented feedback, while motor learning was associated with comparatively permanent changes in performance after removing the feedback and allowing time for memory consolidation (Williams and Carnahan, 2014). Finally, we consider *transfer* as the generalizability of performance improvement that is assessed on an altered version of the trained nominal task.

Motor learning was assessed by so-called retention tests, where the goal task was executed without feedback. Retention tests that were conducted in the same day as the training trails were called short-term retention tests, whereas the tests that were administered at least 24 h after the training trails were called long-term retention tests. In addition, to evaluate generalization of learning, we conducted three transfer tests.

In this study, motor learning and transfer for each participant was evaluated in terms of movement accuracy and consistency. Accuracy and consistency were determined by dissimilarity metrics of error and variability, respectively. Error and variability dissimilarities were calculated for spatial and velocity aspects of the task, resulting in a total of four outcome metrics: spatial error (ε_s_), spatial variability (ν_s_), velocity error (ε_v_), velocity variability (ν_v_).

In general, error is defined as the dissimilarity to the reference movement, while the variability is defined as the dissimilarity within participant's own movements (Gerig et al., 2017, 2019). The spatial error was calculated from the average deviation of participant's stroke trajectories from the reference trajectory. The spatial variability was calculated from the average deviation of participant's each stroke trajectory from his/her other stroke trajectories in the same training or test run. Similarly, velocity error, and variability were calculated from the average deviation of participant's stroke velocity profile from the reference velocity and his/her other stroke velocity profiles in the same run, respectively. Higher error and variability values were associated with lower accuracy and consistency, respectively.

### Data Recording, Data Processing, and Kinematic Evaluation

Kinematic evaluation was based on the recorded vertical (δ) and horizontal (θ) oar angles of the oar blade movement at 100 Hz. In the Simulink® model of the robot, direct kinematics was applied to the measured length of each tendon of the parallel robot to determine the end effector position (*x*_*ee*_). From *x*_*ee*_, coordinate transformation was applied to calculate the angles employed for evaluation (Rauter et al., 2010). Custom-written programs in Matlab® (MathWorks, MA, USA) were used to process and evaluate the data offline.

In the first step of data processing, individual blocks that correspond to instruction, test and training runs of the protocol were isolated. Recorded oar angles were merged to define the rowing cycles based on the smallest θ angle at the beginning of catch phase. For each block, the first three and the last rowing cycles were excluded in order not to include the transition effects from inactivity to rowing and vice versa. Since the rate of reference stroke was 24 spm (i.e., 2.5 s for each cycle) and the participants were instructed to row in the range of 22–26 spm in the test runs, any recorded rowing cycle beyond this range were removed from analysis. Then, both the reference and the valid recorded strokes were resampled to 250 data points for kinematic evaluation.

Processed oar angles were used to evaluate the performance accuracy and consistency in terms of spatial and temporal aspects of the rowing stroke. Any other kinematic aspects were not considered since the participants were only instructed to pay attention to spatial and temporal aspects of the task when reproducing the reference movement. Any further combination of spatial and temporal aspects were also discarded due to lack of information regarding the weight of one against the other.

Evaluation of the error and variability in terms of spatial and velocity aspects were done with dynamic time warping (DTW) (Giese and Poggio, 2000). In the comparison of two time series that have different durations, DTW prevents overemphasizing the spatial error that can arise due to temporal shifts. DTW compares the two time series by employing a cost function that minimizes spatial error and temporal shifts (Vlachos et al., 2003). In this study, the weight of the temporal shift was set to zero. Thus, spatial dissimilarity could be calculated by minimizing the distance between corresponding samples from the participant's one stroke trajectory to the reference trajectory (i.e., spatial error) or participant's other stroke trajectories (i.e., spatial variability) while assuring the causal temporal order of the samples.

Same procedure was also conducted for the evaluation of the velocity dissimilarity, for which the velocity profile at each stroke was compared to the velocity profile of the reference stroke (i.e., velocity error) and other rowing strokes (i.e., velocity variability).

### Statistical Analysis

For the statistical analysis of the four outcome metrics, both Matlab® 2017a and RStudio (Integrated Development Environment for R, version 1.1.463, R Core Team, 2013) were used. The dependent variables were chosen as the spatial error (ε_s_), velocity error (ε_v_), spatial variability (ν_s_), velocity variability (ν_v_). There were no missing data and the variable density (VD) and fixed density (FD) group sizes were equal.

To check if the groups (VD, FD) significantly differed at baseline tests on Day 1 (BL-d1100 for ρ = 1100 $\frac{kg}{m^{3}}$, BL-d200 for ρ = 200 $\frac{kg}{m^{3}}$, BL-d1800 for ρ = 1800 $\frac{kg}{m^{3}}$ and BL-d3200 for ρ = 3200 $\frac{kg}{m^{3}}$), retention tests on Day 2 (RE2-d1100) and Day3 (RE3-d1100), and transfer tests (TRS-d200, TRS-d1800, TRS-d3200) on Day 3, one-way ANOVA was used for each outcome metric. Univariate normality assumption was checked with Q-Q plots. Levene's test was used for checking the homogeneity of variance. If no homogeneity of variance was present, non-parametric Kruskal-Wallis test was used instead of one-way ANOVA.

To check whether the groups reduced their errors and variability from Day 1 to Day 2 and Day 3, a linear mixed effect (lme) model was constructed as shown in below.

(5) $$DV_{i}\;\sim Group*Time\;+(1\;|\;Participant)$$

where *DV*_*i*_ is the dependent variable and *i* = 1…4 is the index for four outcome metrics; *Group* is a categorical independent variable that has two levels FD and VD; *Time* is also a categorical independent variable defined for the tests included in the lme; and finally *Participant* is the random factor that was used since data from different days belonged to one participant.

The lme model (5) was performed separately to test retention of the nominal task condition (with density C, Table 1) and the generalization of learning in the transfer task conditions (with densities A, D, and F, Table 1). Thus, the levels of Time for the nominal task were defined as Baseline, RE2 and RE3, and the levels for the transfer tasks were Baseline and Transfer for each conditions (A, D, and F) separately.

To check significance, *p*-values of the lme model results were retrieved using the “*lmerTest*” package. For lme model (5), “*anova*” method was used for the main effects of *Group* and *Time* and their interaction. A follow-up lme model (6) was constructed separately to determine within-group changes in FD and VD for accuracy and consistency metrics.

(6) $$DV_{i}\;\sim \;Time\;+(1\;|\;Participant)$$

*post-hoc* analysis of the lme model (6) was performed with the “*glht*” function from “*multcomp*” package for each group. The *post-hoc* analysis was only necessary for the main nominal task condition (C), since there were three levels (days): *RE*2 − *BL*_*C*_, *RE*3 − *BL*_*C*_ and *RE*3 − *RE*2. For the transfer conditions with A, D and F, the “*summary*” method for lmer model was sufficient to check for differences of *TRS*_*A*/*D*/*F*_− *BL*_*A*/*D*/*F*_. Multiple comparisons were corrected with Tukey method. The normality of the residuals from the lme models were inspected with Q-Q plots.

*p*-values below 0.05 were considered to show significance. In addition, *p*-values that are between 0.05 and 0.1 were presented as “trending toward significance.”

## Results

### Differences Between Groups at Baseline on Day 1

One-way ANOVA on BL-d1100 showed a trending difference for spatial error [*F*(1, 14) = 4.12, *p* = 0.062]. No significance was revealed for any variables for BL-d200. One-way ANOVA on BL-d1800 showed that VD performed better than FD with a trending difference for spatial error [*F*(1, 14) = 3.91, *p* = 0.068] and significant difference in velocity variability [*F*(1, 14) = 8.84, *p* = 0.01]. The VD group had a significantly smaller velocity variability than FD during BL-d3200 [*F*(1, 14) = 14.47, *p* = 0.002].

### Differences Between Groups at the Long-Term Retention Tests on Day 2 and Day 3

One-way ANOVA showed a significant difference for spatial variability [*F*(1, 14) = 5.83, *p* = 0.03] and trending difference for velocity variability [*F*(1, 14) = 3.56, *p* = 0.08] in favor of VD. However, Levene's test indicated unequal variances for velocity variability [*F*(1, 14) = 7.24, *p* = 0.018], and non-parametric Kruskal-Wallis test did not reveal any significance. One-way ANOVA found a significant difference for spatial variability [*F*(1, 14) = 8.23, *p* = 0.012] in favor of VD.

### Differences Between Groups at the Transfer Tests on Day 3

One-way ANOVA showed that VD reached significantly lower values than FD for spatial variability in all transfer tests [TRS-d200: *F*(1, 14) = 6.89, *p* = 0.02; TRS-d1800: *F*(1, 14 ) = 7.71, *p* = 0.015; TRS-d3200: *F*(1, 14) = 9.73, *p* = 0.008]. A trending difference was also shown for velocity variability at TRS-d200 [*F*(1, 14) = 4.02, *p* = 0.064].

### Learning From Baseline to the Retention Tests for d1100

#### Fixed Effects

The LME model (5) did not show any significant *Group* × *Time* interaction except for spatial error, which was in favor of FD (Table 2). Main effect of *Time* (BL-d1100, RE2-d1100, RE3-d1100) was significant for all outcome metrics, showing a decrease in the value of all main outcome variables from baseline to the third day. The LME model revealed a significant main effect of *Group* for spatial variability and a trending main effect of *Group* for velocity variability, i.e., VD group's mean values were lower than FD group in all days.

**Table 2:** **Group** × **Time** interaction, main effects of **Group** (VD, FD) and **Time** (BL-d1100, RE2-d1100, RE3-d1100) are shown on the left column.

**Variable**	**Interaction and main effects of group and time**		**Within FD group**		**Within VD group**	
	**Group (2) × Time (3)**	**p**	**Effect of Time**	**p**	**Effect of Time**	**p**
	**Group**		***post-hoc***		***post-hoc***	
	**Time**					
ε_s_			*F*_*t*_ (2, 16) = 14.24	**<0.001**	*F*_*t*_ (2, 16) = 16.03	**<0.001**
						
	*F*_*gt*_ (2, 32) = 3.84 *F*_*g*_ (1, 16) = 2.93 *F*_*t*_ (2, 32) = 25.29	**0.032** 0.106 **<0.001**	BL to RE2 BL to RE3 RE2 to RE3	** <0.001** **<0.001** 0.988	BL to RE2 BL to RE3 RE2 to RE3	**0.001** **<0.001** **0.026**
ε_v_			*F*_*t*_ (2, 16) = 8.67	**0.003**	*F*_*t*_ (2, 16) = 6.71	**0.007**
	*F*_*gt*_ (2, 32) = 1.40 *F*_*g*_ (1, 16) = 0.06 *F*_*t*_ (2, 32) = 14.36	0.261 0.815 **<0.001**	BL to RE2 BL to RE3 RE2 to RE3	**0.002** **<0.001** 0.558	BL to RE2 BL to RE3 RE2 to RE3	0.171 **<0.001** **0.048**
ν_s_			*F*_*t*_ (2, 16) = 5.38	**0.016**	*F*_*t*_ (2, 16) = 11.88	**<0.001**
						
	*F*_*gt*_ (2, 32) = 0.35 *F*_*g*_ (1, 16) = 5.99 *F*_*t*_ (2, 32) = 16.23	0.704 **0.026** **<0.001**	BL to RE2 BL to RE3 RE2 to RE3	**0.043** **0.004** 0.381	BL to RE2 BL to RE3 RE2 to RE3	**0.003** **<0.001** 0.107
						
ν_v_			*F*_*t*_ (2, 16) = 3.29	0.064	*F*_*t*_ (2, 16) = 19.36	**<0.001**
						
	*F*_*gt*_ (2, 32) = 1.39 *F*_*g*_ (1, 16) = 3.57 *F*_*t*_ (2, 32) = 5.82	0.264 0.077 **0.007**	BL to RE2 BL to RE3 RE2 to RE3	0.076 0.058 0.791	BL to RE2 BL to RE3 RE2 to RE3	**<0.001** **<0.001** 0.24

*Follow-up LME and post-hoc within each group for main effect of Time are shown in the middle (FD group) and on the right column (VD group). Significant differences (p < 0.05) were highlighted with bold*.

#### LME Within Each Group

The LME model (6) showed a main effect of *Time* for both groups and all metrics except velocity variability for FD group, which showed only a trending effect (see Table 2).

*Learning from BL to RE2:* FD significantly reduced mean values for all metrics except velocity variability. VD showed a significant decrease for all metrics except velocity error.

*Learning from BL to RE3:* Both groups significantly improved in all metrics except the velocity variability in FD group.

*Learning from RE2 to RE3:* FD did not show any significant decrease in any of the metrics while VD significantly reduced spatial and velocity mean errors.

## Discussion

In this study, the effects of a randomized order of variable density training and a fixed density training were investigated on a real-life complex motor task. We hypothesized that training with the variable density training protocol would result in a superior learning and generalization when compared to the fixed density training due to the high contextual variability and potential increase of functional task difficulty provided to the participants. Both learning and generalization were assessed in terms of accuracy and consistency, which are among the main features of motor skill development according to Wulf (2007); Schmidt and Wrisberg (2008). Accuracy and consistency were measured by spatial and velocity aspects of the reference movement, resulting in a total of four outcome metrics: spatial error, velocity error, spatial variability, and velocity variability. In the following subsections, results of each outcome metric are discussed.

### Spatial Error

**Figure 4:**
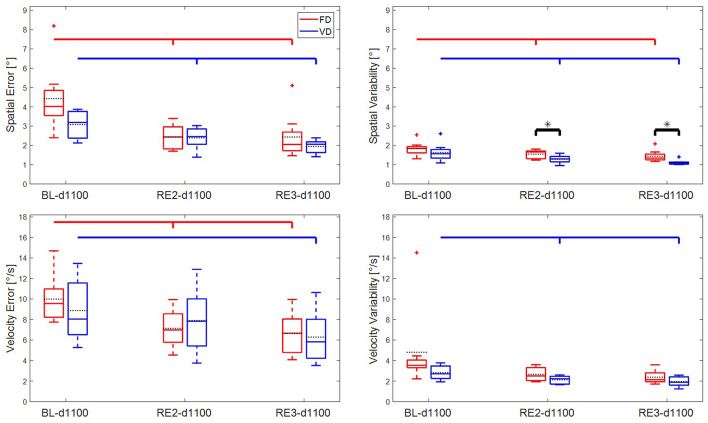
Main outcome metrics vs. main condition tests for all subjects of the two groups: spatial error **(Upper Left)**, spatial variability **(Upper Right)**, velocity error **(Lower Left)**, and velocity variability **(Lower Right)**. The boxes in group color denote median and the edges of the box are the 25 and 75th percentiles. Group means are shown by black dashed line. The whiskers denote ±2.7 standard deviations or 99.3% coverage intervals. Plus (+) symbols shown in the corresponding group colors indicate the outliers. Black starred (*) bars illustrate the main effect of group in the respective test run. The main effect of Time within each group are indicated by horizontal lines that are shown in the corresponding group color. Significant differences from BL-d1100 to RE2-d1100 and BL-d1100 to RE3-d1100 are denoted by vertical marks below the horizontal lines.

**Figure 5:**
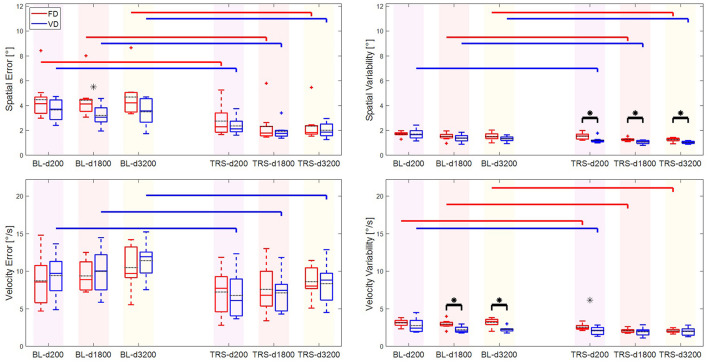
Main outcome metrics vs. transfer condition tests for all subjects of the two groups: spatial error **(Upper Left)**, spatial variability **(Upper Right)**, velocity error **(Lower Left)**, and velocity variability **(Lower Right)**. Each density condition is displayed with a background color that corresponds to the water rendering color in visual scenario from Table 1.

Both groups were able to significantly reduce the spatial error from baseline to the final tests in the third day (see Figures 4, 5). Although the VD group's mean spatial error was lower than FD at RE3-d1100, FD group reduced spatial error more than VD over three days [*F*_*gt*_ (2, 32) = 3.84, *p* = 0.032, see Table 2]. This significant *Group* × *Time* interaction was probably due to the fact that the FD group started with a worse performance (trending difference, see Figure 4) than the VD group at BL-d1100 test.

The performance difference between the groups in the BL-d1100 and significant *Group* × *Time* interaction obstructs making a comparison regarding the benefit of each training method for naïve participants. The progress of FD group may suggest that provision of fixed practice conditions with visual feedback training and KR was beneficial for initially less skilled beginners. Additionally, initially more skilled beginners might have benefited from the provided variable practice (significant effect of *Time* for both groups, see Figure 4). Based on the significant *Group* × *Time* interaction, we may speculate that for the given initial skill level in both groups, fixed practice suited FD group more than variable practice suited VD group.

Lacking significant *Group* × *Time* interaction in any of the transfer test conditions implies that both groups could reduce their mean spatial errors at a comparable rate from the first day baseline tests, although there was a trending group difference at BL-d1800. In all tests, VD group's mean spatial error was less than the FD group, suggesting that the differences in baseline performances between the groups were preserved through the learning process.

Results of spatial error supports the finding that spatial information is mainly perceived visually (Welch and Warren, 1980; Nesbitt, 2003). In literature, comparable studies showed that addition of reactive haptic feedback for a complex rowing (Sigrist et al., 2015), path control feedback for a 2D shape drawing (Yang et al., 2008) and haptic guidance in Chinese handwriting (Xiong et al., 2013) tasks did not result in a significantly better learning than visual alone in the delayed retention tests. In our study, participants did not receive any haptic feedback during the training sessions. The robotic simulator only haptically rendered different water conditions on top of the displayed visual feedback. Both groups' comparable progress of learning from baseline to the third day tests may suggest that presence of visual feedback rather than the haptically modulated task-inherent conditions contributed to the overall development of spatial accuracy. Thus, previous statements suggesting that the visual perception plays a key role for spatial information could also be confirmed with a comparison to the haptic rendering of task characteristics.

Presumed ineffectiveness of additional haptic information can also be supported by inspecting the VD group's spatial accuracy during the training sessions (see Figure S1 for spatial error). In the first day of training, both groups could reduce their spatial error, which can be attributed to overall familiarization with the task, which was also observed in our previous studies sharing a similar protocol (Rauter et al., 2015; Sigrist et al., 2015; Gerig et al., 2019). In the second day of training, FD group showed a ceiling effect considering the lack of change in the mean spatial error values. On the other hand, VD group's mean spatial errors varied in each training but the change was subtle. This result may imply that the visual feedback alone could not help FD group further reduce error values; but the additional variable task-inherent haptics were also ineffective to result in a statistically lower spatial mean error values in the VD group.

### Velocity Error

Significant *Time* effect for each VD and FD groups over the main condition (d1100) tests suggests that both groups reduced the velocity error from BL-d1100 to RE3-d1100. In BL-d1100, both groups started at a comparable level of velocity error; however, the VD group could not significantly reduce their mean error to the second day retention test (RE2-d1100, see Figure 4). Although this result might seem contrary to the hypothesis, it is not surprising because of the nature of the training that VD received. According to the variability of practice hypothesis (Schmidt, 1975), failure of acquiring the given task through variable practice suggests that the required corresponding motor schemata may have not been successfully formed. If the learner is in the early stage of skill development, less practice variability might provide more optimal information (Guadagnoli and Lee, 2004).

The inability to reduce velocity error in the early motor development stage may also be explained by the randomized design of the provided training. Compared to blocked design, randomized training yields higher functional task difficulty (Guadagnoli and Lee, 2004) and cognitive activity that is explained by both the elaboration hypothesis (Shea and Morgan, 1979; Shea and Zimny, 1983; Wright et al., 1992) and the reconstruction hypothesis (Lee and Magill, 1983, 1985). Increased task difficulty and cognitive activity might have been too demanding if the VD group could not proportionally advance the skill level in the first day. However, the significant velocity error reduction from RE2-d1100 to RE3-d1100 implies that VD group could benefit from the randomized training and was able to overcome the initially too demanding cognitive effort.

In agreement with our hypothesis, only VD group significantly reduced the velocity error from baseline to all transfer tests on the third day (see Table 3 and Figure 5). FD group did not train the task under different density conditions, which might have provided crucial temporal information to transfer the retained skill of dealing with different haptic requirements. In literature, the effect of contextual interference has been shown to result in a more enhanced transfer performance than practicing the same condition in the trainings (Merbah and Meulemans, 2011). In a study, effect of different haptic training strategies for a simple virtual ball bouncing task under various gravity conditions was investigated (Marchal-Crespo et al., 2014b). The authors stated that the training strategy that allowed for an enriched task experience also improved the spatiotemporal accuracy of the group in the untrained (transfer) gravity conditions. In another study that explored the robotic guidance effect on a simple pinball-like simple hitting game, researchers found that the training strategy, which limited the variety of overall training, did not benefit the temporal accuracy in transfer tests (Marchal-Crespo and Reinkensmeyer, 2008b). Thus, the advantage of haptically presented practice variability for improving the temporal accuracy in transfer tests could also be confirmed and extended to a complex sports skill.

**Table 3:** **Group** × **Time** interaction, main effects of **Group** (VD, FD) and **Time** (BL-d200 to TRS-d200; BL-d1800 to TRS-d1800, BL-d3200 to TRS-d3200) are shown on the left column for each density condition.

**Condition**	**Variable**	**Interaction and main effects of group and time**		**Within FD group**		**Within VD group**	
		**Group (2) × Time (2)**	**p**	**Effect of Time**	**p**	**Effect of Time**	**p**
		**Group**		***post-hoc***		***post-hoc***	
d200	ε_s_	*F*_*gt*_ (1, 16) = 0.611 *F*_*g*_ (1, 16) = 2.07 *F*_*t*_ (1, 16) = 12.66	0.610 0.161 **0.001**	*F*_*t*_ (1, 8) = 5.29 BL to TRS	**0.037** **0.037**	*F*_*t*_ (1, 8) = 13.33 BL to TRS	**0.006** **0.006**
	ε_v_	*F*_*gt*_ (1, 16) = 1.32 *F*_*g*_ (1, 16) = 0.01 *F*_*t*_ (1, 16) = 16.78	0.268 0.930 ** <0.001**	*F*_*t*_ (1, 8) = 3.69 BL to TRS	0.091 0.091	*F*_*t*_ (1, 8) = 16.72 BL to TRS	**0.003** **0.003**
	ν_s_	*F*_*gt*_ (1, 16) = 4.01 *F*_*g*_ (1, 16) = 2.46 *F*_*t*_ (1, 16) = 16.85	0.062 0.136 ** <0.001**	*F*_*t*_ (1, 8) = 3.46 BL to TRS	0.099 0.099	*F*_*t*_ (1, 8) = 13.69 BL to TRS	**0.006** **0.006**
	ν_v_	*F*_*gt*_ (1, 16) = 0.14 *F*_*g*_ (1, 16) = 2.81 *F*_*t*_ (1, 16) = 15.86	0.710 0.113 **0.001**	*F*_*t*_ (1, 8) = 7.15 BL to TRS	**0.016** **0.016**	*F*_*t*_ (1, 8) = 9.26 BL to TRS	**0.016** **0.016**
d1800	ε_s_	*F*_*gt*_ (1, 16) = 0.96 *F*_*g*_ (1, 16) = 3.78 *F*_*t*_ (1, 16) = 16.55	0.335 0.062 ** <0.001**	*F*_*t*_ (1, 8) = 7.93 BL to TRS	**0.014** **0.014**	*F*_*t*_ (1, 8) = 23.45 BL to TRS	**0.001** **0.001**
	ε_v_	*F*_*gt*_ (1, 16) = 1.01 *F*_*g*_ (1, 16) = 0.003 *F*_*t*_ (1, 16) = 18.97	0.330 0.956 ** <0.001**	*F*_*t*_ (1, 8) = 4.06 BL to TRS	0.078 0.078	*F*_*t*_ (1, 8) = 23.24 BL to TRS	**0.001** **0.001**
	ν_s_	*F*_*gt*_ (1, 16) = 0.31 *F*_*g*_ (1, 16) = 3.30 *F*_*t*_ (1, 16) = 24.06	0.583 0.088 ** <0.001**	*F*_*t*_ (1, 8) = 7.01 BL to TRS	**0.029** **0.029**	*F*_*t*_ (1, 8) = 22.82 BL to TRS	**0.001** **0.001**
	ν_v_	*F*_*gt*_ (1, 16) = 7.29 *F*_*g*_ (1, 16) = 5.53 *F*_*t*_ (1, 16) = 25.51	**0.016** **0.032** **0.001**	*F*_*t*_ (1, 8) = 24.30 BL to TRS	**0.001** **0.001**	*F*_*t*_ (1, 8) = 3.62 BL to TRS	0.094 0.094
d3200	ε_s_	*F*_*gt*_ (1, 31.9) = 1.05 *F*_*g*_ (1, 31.9) = 3.38 *F*_*t*_ (1, 31.9) = 21.11	0.313 0.075 ** <0.001**	*F*_*t*_ (1, 8) = 9.27 BL to TRS	**0.009** **0.009**	*F*_*t*_ (1, 8) = 21.31 BL to TRS	**0.002** **0.002**
	ε_v_	*F*_*gt*_ (1, 16) = 1.25 *F*_*g*_ (1, 16) = 0.09 *F*_*t*_ (1, 16) = 22.10	0.280 0.761 ** <0.001**	*F*_*t*_ (1, 8) = 4.58 BL to TRS	0.065 0.065	*F*_*t*_ (1, 8) = 28.29 BL to TRS	** <0.001** ** <0.001**
	ν_s_	*F*_*gt*_ (1, 16) = 0.12 *F*_*g*_ (1, 16) = 5.26 *F*_*t*_ (1, 16) = 30.72	0.739 **0.036** ** <0.001**	*F*_*t*_ (1, 8) = 10.80 BL to TR	**0.011** **0.011**	*F*_*t*_ (1, 8) = 23.14 BL to TRS	**0.001** **0.001**
	ν_v_	*F*_*gt*_ (1, 16) = 17.88 *F*_*g*_ (1, 16) = 6.95 *F*_*t*_ (1, 16) = 49.43	** <0.001** **0.018** ** <0.001**	*F*_*t*_ (1, 8) = 81.80 BL to TRS	** <0.001** ** <0.001**	*F*_*t*_ (1, 8) = 3.20 BL to TRS	0.111 0.111

*Follow-up LME and post-hoc within each group for main effect of Time are shown in the middle (FD group) and on the right column (VD group). Significant differences (p < 0.05) were highlighted with bold*.

In our study, the effect of the modulated task difficulty can also be seen from the variation of VD group's performance during training (see Figure S1 for velocity error). VD group's mean velocity error during training was dependent on the presented density condition. In general, larger and lower mean error values were associated with training in a higher and lower density conditions, respectively. Presence of different training conditions contributed to an enhanced range of task experience, which yielded different level of mean velocity error values. On the contrary, FD group could reduce velocity errors until TR4, but the changes in the mean velocity error values between the following trainings were less pronounced compared to VD group. However, this outcome cannot be due to the lack of motivation. The IMI questionnaire results from the training reveal that both groups reported similar levels of competence, interest, effort and usefulness (see Figure S2). Therefore, we may associate the long-term improvement of velocity accuracy of VD group with the overall increased task related information due to variable density training.

### Spatial Variability

The effect of variable training was most prominent on the spatial variability metric. Although *Group* × *Time* interaction was not significant and both groups managed to significantly reduce spatial variability from the baseline to the retention and transfer tests (except FD group for d200 condition, see Tables 2, 3), the VD group reached significantly lower variability than FD in all tests (see Figures 4, 5). The result from retention tests is especially remarkable given the fact that VD group only trained in this specific condition only one third of the time compared to FD.

The benefit of the variable training on the spatial variability can be attributed to the effect of attentional focus (Wulf and Shea, 2002). Due to the design of visual feedback in training sessions, participants usually looked at the oar blade and the traces drawn on the right screen. However, the exposure to modulated task dynamics in addition to the required task might have made the VD participants focus on the “effect of the movement of the oar” (external focus) rather than the “movement of the oar itself” (internal focus). Thus, the external focus may have yielded more enhanced learning as reported in Wulf et al. (2010); Wulf (2013).

In addition, the promoted adoption of the external focus may have resulted in implicit learning (Maxwell et al., 2001). The implicit learning occurs when the motor skill develops without the explicit knowledge related to the given task. In these cases, learners may perform the task by adopting an external focus, which can restrict the conscious thinking about task, resulting in implicit learning (Johnson, 2014). In our study, neither of the groups received additional information regarding their variability. Prior to each test, both groups were instructed to replicate the reference movement as accurately as possible based on what they remember (or learn) from the training session. After each test, the performance chart showed only the progress of spatial and velocity errors, which is independent of the variability. Thus, in the absence of explicit information related to variability, concentration on the effect of movement might have guided VD group to implicitly adopt an own way and maintain it to cope with the “disturbance” caused by changing haptics.

### Velocity Variability

The variable training scheme was effective on the velocity variability for the retention tests (see Figure 4). Although *Group* × *Time* interaction was not significant and both groups could reduce their variability from baseline to retention, *post-hoc* analysis showed that variability reduction was significant within VD group from BL-d1100 to RE3-d1100, but learning from RE2-d1100 to RE3-d1100 did not occur.

This result is in line with the progress of velocity variability from training session (see Figure S1). In the first day trainings (TR1 to TR6), VD group's variability sharply decreases until TR4, while the FD shows rather moderate but continuous reduction. Similar to the relation observed between the velocity error and training condition, the change of density affected the velocity variability, but in a less pronounced magnitude. As explained in the spatial variability metric, implicit learning due to the external focus effect might also have played a role in the significant reduction of velocity variability.

In the second day trainings, both groups showed a plateau effect, suggesting that the provided information could not support further skill development. This result is interesting because we would have expected from VD group a further progress on the precision based on the previously reported effectiveness of practice variability (Donakowski, 2005) and randomized training (Ali et al., 2012) on the temporal variability. However, these studies investigated artificial laboratory tasks to be learned; thus, conclusions from simple tasks may not extend to the real-life complex tasks (Wulf and Shea, 2002). Nevertheless, we can deduce that the amount of total information (i.e., task difficulty) was not optimal to allow the VD group to progress in terms of velocity precision during the second day.

The effect of possibly sub-optimal task difficulty related to velocity variability was also seen in the transfer tests. Both groups could significantly reduce variability over time for d200 condition and a trending difference (*p* = 0.065) occurred between the group means at TRS-d200. However, VD group could not show any significant reduction of velocity variability for d1800 and d3200 conditions, for which FD group significantly reduced variability from corresponding baseline tests.

The main reason of insignificant effect of variable training on the velocity variability may actually be due to the significant differences between groups at the BL-d1800 and BL-3200 (see Figure 5). Although VD group showed similar baseline performances for d200 and d1100 tests, the velocity variability was lower for d1800 and d3200 tests. VD group performed already very advanced compared to FD in both BL-d1800 and BL-d3200. Therefore, if the functional task difficulty was not sufficient for the skill level of VD group, they could not benefit from the little available potential information to progress more (Guadagnoli and Lee, 2004).

### General Remarks

In literature, variable practice was found to be beneficial for motor learning of simple laboratory tasks (Shea and Kohl, 1990; Donakowski, 2005; Huang et al., 2009) and real-life sports tasks (Shoenfelt et al., 2002; Bartlett et al., 2007) as well as for the robot-assisted training on simple tasks (Duarte and Reinkensmeyer, 2015; Agarwal and Deshpande, 2017). In this study, previous findings regarding the benefit of variable practice in separate domains could be successfully merged for the learning of a real-life complex task with a robotic simulator.

The overall significance of the variable training may be argued to be subtle by the critics. However, it should be noted that both groups received the concurrent augmented visual feedback in the training sessions and KR about the mean spatial and velocity error values after the tests. In our previous studies (Rauter et al., 2015; Sigrist et al., 2015; Gerig et al., 2019), visual feedback was found to be the most effective feedback to assist learning of both reference spatial and temporal characteristics of the rowing task. Thus, the FD group was not a conventional control group, who did not receive any reference task related information during training. In such a case, the contrast between the groups would be maximal since the control group receiving no feedback would very likely learn nothing. Instead, we showed that although the provision of visual feedback and KR was already very effective, introduction of variable density training have resulted in a superior spatial consistency and velocity accuracy in both retention and transfer tests.

In this study, we assumed that each change in the resistive task forces due to the modulation of density contains a certain amount of challenge for learning the task. However, the perceived challenge might have differed according to learner's skill level. Although the challenge point framework suggests to optimally adapt the challenge, an existing knowledge on how to tailor the modulation of the density conditions to each individual was unavailable to us. Thus, we assumed that randomized presentation of different conditions as a training block would yield a certain amount of increment in the functional task difficulty for VD group. This increased amount of information resulted in different rates of learning and generalization for each metric, partially due to participants' varying initial skill level and ability to progress in the respective movement aspects (see Figures 4, 5). In addition, the level of increased difficulty may have stayed constant from one training block (randomized order of three different density conditions, e.g., TR1-TR2-TR3) to another. Thus, the amount of learning was also different in the first and second day. Naïve participants of VD group could mostly benefit from the available information in the first day to reduce mean values for all metrics except velocity error (BL to RE2, see Table 2). However, comparable amount of information received from second day's training might have been sub-optimal for VD group to promote the learning at the same rate for variability metrics at retention (RE2 to RE3, see Table 2). Therefore, researchers investigating the impact of “practice variability” and “practice schedule” (random vs. blocked) on motor learning are recommended to take into account the amount of available information to participants in all of the trainings when interpreting their results for a complex task.

In general, although the increased functional task difficulty due to variable practice helped participants to learn the task, it would be desirable that the challenge is adapted according to the skill development of the learner on a certain aspect of the task. In addition, the overall task difficulty was assumed to be affected by only modulating the “water density” task parameter. The modulation may have actually had different effects on each independent outcome metric. For example, provision of different task-inherent forces might have increased the functional task difficulty and cognitive activity more for achieving the velocity accuracy than for the spatial accuracy. Instead, it may be more promising that the researchers in motor learning field: (I) choose a primary aspect of motor task (e.g., spatial, temporal, spatiotemporal error or variability) to be learnt, (II) find a relevant task-inherent parameter to directly modulate the difficulty, (III) measure a related outcome metric (e.g., angular deviation from a reference for spatial; speed for temporal; velocity for spatiotemporal aspect) to adapt the available information according to the skill development of the learner. Similar procedures were previously employed in the studies that investigated the effect of augmented haptic feedback methods. Instead of modulating the task-inherent parameter to adapt difficulty, researchers measured the outcome metrics to adapt the external forces acting on the task during training for the rowing task (Rauter et al., 2015) and a locomotor task (Marchal-Crespo et al., 2014a) and in between the training trials (Marchal-Crespo and Reinkensmeyer, 2008a) for a steering task.

In literature, the majority of the investigated research regarding the contextual variability were done on discrete skills, i.e., short in duration and incorporating distinct beginning and end (Schmidt and Wrisberg, 2008). A few laboratory studies focusing on continuous skills, i.e., incorporating relatively long task execution, such as rotary pursuit (Whitehurst and Del Rey, 1983) and a continuous bimanual coordination task (Smith, 1997) could not previously show the effectiveness of the contextual variability (Merbah and Meulemans, 2011). In our study, we found that the contextual variability could indeed benefit motor learning and generalization of a real-life continuous task such as rowing (Marchal-Crespo et al., 2015). This result may suggest that the optimality of the challenge resulted from variability plays an important role to support learning, which may have been missing in the previous studies that investigated continuous skills.

In addition to the applicability of findings of our study to other similar sport skills, e.g., kayaking, canoeing, cycling or running, the current results may also have implications for robot-assisted locomotor rehabilitation. In the field of gait rehabilitation with robotic devices, many research teams attempted to apply assistive and/or resistive forces (Dong et al., 2005; Lam et al., 2015; Mun et al., 2017; Wu et al., 2017; Marchal-Crespo et al., 2019) to the leg movements to restore the gait function of patients who suffered from spinal cord injury and stroke. Since the adjustment of task difficulty in the training conditions can influence the effectiveness of the movement restoration (recovery) process (Kizony et al., 2003), it is important to devise suitable intervention protocols. In the cases where there is no explicit knowledge on how to adjust the task-inherent parameters to the recovery rate of patient, researchers may apply the findings from practice variability to explore the condition effects and the optimality of the provided task difficulty.

## Limitations

A technical limitation related to our rowing simulator might have hampered the effectiveness of haptically presented practice variability for spatial error reduction for VD group. The modulation of density mainly affected the rendered drag and lift forces of the water in the rowing simulator. Due to this effect, we considered that participants would need to pay more attention for manipulating the oar in low-density conditions compared to high-density conditions since they may easily overshoot from the reference trajectory due to reduced damping. However, especially at the release phase of the rowing cycle, there were unwanted friction forces due to the technical design of the tendon-based parallel robot, which may have interfered with the desired task forces. When the protocol was over, a few participants verbally informed us about the struggle they experienced at the release. Therefore, the undesired forces might have unintentionally distracted the participants and decreased the effectiveness of variable training for improving spatial accuracy.

In general, differences between the groups at the baseline tests due to inter-subject variability are not desirable since it may bias the statistical analysis and affect the results (Roberts and Torgerson, 1999). This could be prevented by distributing the participants based on their baseline test performances (Patoglu et al., 2009; Marchal-Crespo et al., 2010) or providing both groups the same intervention procedure after which they could potentially reach to similar performances (Gerig et al., 2019). The former approach is promising if all the outcome metrics could be assessed in an online manner with reasonably chosen threshold values. In our study, we could assess both spatial and velocity error during the tests but variability dissimilarity had to be processed offline due to computational complexity, which would require a certain amount of waiting time. According to our study protocol, the first day training session was executed right after the baseline tests, for which we did not have enough time to inspect all metrics for all different baseline tests. The latter approach taken in Gerig et al. (2019) is also effective in terms of reaching to a certain baseline equality; however, the participants would not be “naïve” anymore. In our study, since the aim was to support learning from early to late motor skill stages, we targeted naïve participants.

The choice of training strategy and the protocol design might also have influenced the learning and generalization of velocity accuracy. The randomized order of variable density training might have been sub-optimal for the given complexity of the task. For complex tasks that are difficult to learn, literature suggests to employ a blocked practice which can be followed by a randomized one to allow an efficient learning process (Wulf and Shea, 2002). Lastly, our training protocol was limited to 2 days of training in total. Although VD groups continued to reduce the velocity error during training performances, we cannot speculate how they would have progressed had we had a longer protocol.

## Conclusion

In this study, we found that the provision of practice variability by means of haptically modulated density conditions was beneficial for learning and generalization of spatial as well as temporal aspects of a real-life rowing task. Robotically introduced training variability showed a potential to be more beneficial than the combination of KR and concurrent augmented visual feedback, which has earlier been found to be the most effective feedback for our complex rowing skill. Thus, the results indicate that for a given training period, practicing the kinematic and dynamic variations of a target task can be more advantageous than repeatedly attempting the task itself. Practice variability was found to be especially useful for enhancing the task execution consistency, which is an important dimension in skill development.

To the best of our knowledge, we were the first group to inspect the effect of practice variability on a complex sports skill with a robotic simulator. Thus, the findings from the study are especially important for the employment of robotic systems for supporting learners acquire new motor skills or recover from impaired motor abilities. That is because, despite the popularity of robot-assisted training in many fields, the main concern remains as the application of training methods that can optimally support skill development. Therefore, current findings demonstrate that motor learning can be assisted from early to further motor skill stages by means of modulating the challenge applied during the trainings on a robotic system.

In this study, although the increased functional task difficulty due to practice variability supported learning in general, it was not adapted to the skill development of the participants. To avoid plateau effects on the movement aspects and assist learning even more optimally, the challenge presented to the participants needs to be modulated in an automated way. Since we could measure the effect of the changes between the density conditions on the performance metrics during training, recorded data will be helpful to design future studies in which we seek to adapt the challenge to each individual.

## Data Availability

The datasets generated for this study are available on request to the corresponding author.

## Author Contributions

EB contributed to the rowing model development, graphical user interface design, experimental protocol design, IMI questionnaire preparation, conducting the experiments and data acquisition, kinematic evaluation, statistical analysis and prepared the manuscript. LM-C participated in the experimental protocol design, IMI questionnaire preparation and revision of the manuscript. GR contributed to the solution of technical problems encountered during rowing model development. RR participated in the overall discussion of results and revision of the manuscript. PW participated in the experimental protocol design, IMI preparation, contributed to discussion regarding the statistical analysis and revised the manuscript.

### Conflict of Interest Statement

The authors declare that the research was conducted in the absence of any commercial or financial relationships that could be construed as a potential conflict of interest.
