# Supplementary material for: Rowing Simulator Modulates Water Density to Foster Motor Learning
Source: Front Robot AI. 2019 Aug 21;6:74. doi: 10.3389/frobt.2019.00074 (PMC7806073; doi:10.3389/frobt.2019.00074)
Supplement: Supplementary file 1 [file Table_1.DOCX]

Supplementary Material

Figure S1. Main outcome metrics vs. training trails for all subjects of the two groups: spatial error (upper left), spatial variability (upper right), velocity error (lower left), and velocity variability (lower right). Each density condition is displayed with a background color that corresponds to the water rendering color in visual scenario from Table 1, e.g. blue for C = 1100.

Figure S2. IMI assessment scales during training performance for all subjects of the two groups: Usefulness (upper left), Effort (upper right), Competence (lower left), and Interest (lower right).
